# Insulin Resistance with Associated Hyperinsulinemia as a Cause of the Development and Worsening of Heart Failure

**DOI:** 10.3390/biomedicines12122890

**Published:** 2024-12-19

**Authors:** Umberto Attanasio, Valentina Mercurio, Serafino Fazio

**Affiliations:** 1 Department of Translational Medical Sciences, Federico II University, 80131 Naples, Italy; umberto.attanasio@yahoo.it (U.A.); valemercurio@yahoo.com (V.M.); 2 Department of Internal Medicine, School of Medicine, Federico II University, 80131 Naples, Italy

Despite the considerable progress that has been made with regard to the prevention and treatment of cardiovascular diseases, heart failure (HF) remains a particularly important cause of recurring hospitalizations, with relevant social and healthcare costs, and it is a significant cause of mortality; as such, there is a need for continuous efforts to improve our understanding of its complex pathophysiology and refine relevant therapeutic strategies [1]. The incidence of HF, particularly HF with preserved ejection fraction (HFpEF), continues to rise, partly due to the increased prevalence of insulin resistance (IR) and the subsequent development of type 2 diabetes [2]. This Special Issue helps to shed light on various aspects of HF, with a notable focus on IR’s role in its pathogenesis and the potential benefits of targeting IR to improve HF management.

Researchers are placing increasing emphasis on the intricate interplay between metabolic dysregulation and cardiac function. IR, which is characterized by impaired glucose uptake and utilization by cells, is often accompanied by hyperinsulinemia, a compensatory response aimed at maintaining glucose homeostasis [3,4]. While hyperinsulinemia initially helps regulate blood sugar levels, chronic elevation of insulin exerts detrimental effects on the cardiovascular system, ultimately contributing to HF progression [2]. Several mechanisms appear to link IR/hyperinsulinemia to HF. These include endothelial dysfunction, since IR is thought to interfere in the balance of vasodilating and vasoconstricting factors, leading to impaired endothelial function and increased vascular resistance [3]; left ventricular hypertrophy and remodeling, given that hyperinsulinemia promotes the proliferation of vascular smooth muscle cells and myocardiocytes, resulting in increased left ventricular mass and concentric remodeling (both hallmarks of HFpEF) [4]; myocardial lipid accumulation, occurring as a result of IR-related excess lipid deposition in the myocardium, which interferes with cell signaling and disrupts cardiac structure, leading to lipotoxicity and impaired heart function; and altered myocardial substrate metabolism, since IR shifts myocardial substrate utilization from glucose to free fatty acids, leading to metabolic inflexibility and reduced cardiac function. Furthermore, the association between serum free fatty acid levels and clinical parameters in acute HF patients highlights the complex metabolic dysregulation associated with acute HF, which is often characterized by increased reliance on fatty acid oxidation as an energy source due to impaired glucose utilization [5].

Despite these advances in knowledge, there are still significant gaps in our understanding of IR’s precise role in the various HF subtypes and the optimal strategies for mitigating its adverse effects. This Special Issue addresses some of these gaps by exploring the impact of IR on different aspects of HF pathophysiology and investigating potential therapeutic targets.

The articles published in this Special Issue also offer new potential alternatives for clinical interventions, such as the possible use of point-of-care urine chloride measurement using strip tests as a tool to monitor fluid status and decongestive therapy in patients with HF. Furthermore, the potential therapeutic benefits of targeting IR in HF are explored. The review article discusses the negative impact of IR/hyperinsulinemia on chronic HF and proposes implementing early screening and treatment of IR to improve patient outcomes. The evidence suggests that substances such as metformin and berberine, which are known to reduce IR, may have clinical benefits for HF patients, while others, such as SGLT2 inhibitors and GLP-1 RA, have already proven their value in clinical settings [2]. These findings underscore the importance of considering IR as a modifiable risk factor and therapeutic target in HF management. While the articles in this Special Issue provide valuable contributions to the field, further research is needed to advance our understanding and management of IR in HF. Indeed, future studies should aim to elucidate the specific mechanisms by which IR contributes to the development and progression of various HF subtypes. This will enable the development of tailored therapeutic strategies based on the underlying pathophysiology. Studies should also focus on developing accurate and accessible methods for early IR screening in at-risk individuals and HF patients. Additionally, research is needed to determine the most effective therapeutic agents and optimal dosages for targeting IR in HF, considering potential drug interactions and patient-specific factors. Furthermore, assessing the effectiveness of different dietary approaches, exercise regimens, and combinations of lifestyle interventions in reducing IR and enhancing cardiac function may also provide some valuable information related to preventive medicine. Finally, efforts should focus on identifying and validating new therapeutic targets that specifically address the detrimental effects of IR on the cardiovascular system, with the aim of modulating insulin signaling pathways and improving metabolic flexibility in HF patients.
